# Bioprocess of Microbial Melanin Production and Isolation

**DOI:** 10.3389/fbioe.2021.765110

**Published:** 2021-11-16

**Authors:** Kwon-Young Choi

**Affiliations:** ^1^ Department of Environmental Engineering, College of Engineering, Ajou University, Suwon, South Korea; ^2^ Department of Environmental and Safety Engineering, College of Engineering, Ajou University, Suwon, South Korea

**Keywords:** melanin, pigment, space-time yields, extraction, purification

## Abstract

Melanin is one of the most abundant pigments found in the biosphere. Owing to its high biocompatibility and diverse biological activities, it has been widely applied as a functional biomaterial in the cosmetic, pharmaceutical, biopolymer, and environmental fields. In this study, the production of melanin was comprehensively reviewed concerning bioconversion and isolation processes. First, several melanogenic microbes, including fungi and bacteria, were summarized. Melanin production was classified by host and melanin type and was analyzed by titers in g/L in addition to reaction conditions, including pH and temperature. The production was further interpreted using a space-time yields chart, which showed two distinct classifications in productivity, and reaction conditions were analyzed using a pH-temperature-titer chart. Next, the extraction process was summarized by crude and pure melanin preparation procedures, and the extraction yields were highlighted. Finally, the recent applications of melanin were briefly summarized, and prospects for further application and development in industrial applications were suggested.

## Introduction

Melanin is a representative brown-black pigment commonly found in most organisms. It is widely found in melanin-producing animal cells as well as in bacteria, fungi, and plants. From the black coloration of a human eye, hair, and skin to the black insect epidermis and oxidation-induced discoloration of fruits, melanin occurs in most of the biosphere (Pralea et al., 2019; Singh et al., 2021). Melanin has long been an important component of living organisms and cells. Melanin synthesis in organisms is primarily involved in the protection of host cells and organisms. This includes protection from UV radiation and energy absorption, protection from external physical changes, and maintenance of intracellular homeostasis through its physiological activity (Bolognese et al., 2019; Seo and Choi, 2020a).

**FIGURE 1:**
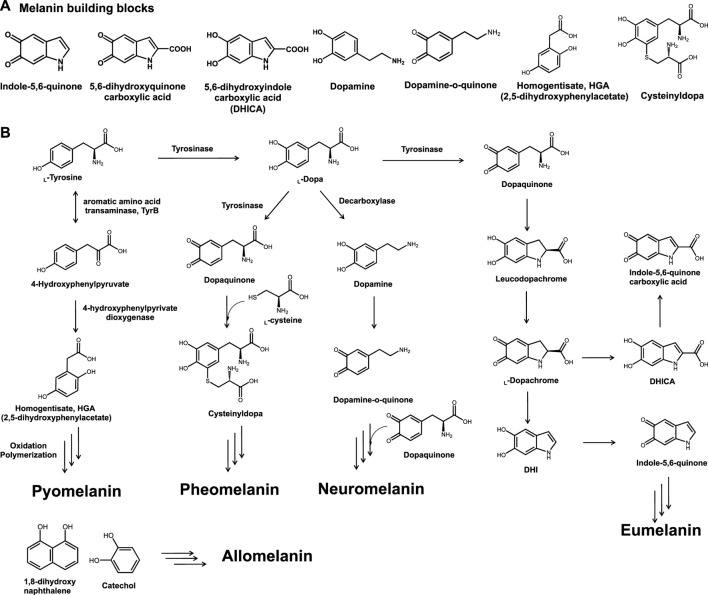
**(A)** Chemical structures of general melanin building blocks, namely, indole-5,6-quinone, 5,6-dihydroxyquinone carboxylic acid, 5,6-dihydroxyindole carboxylic acid, dopamine, dopamine-o-quinone, homogentisate, and cysteinyldopa. **(B)** Types of bio-melanin and synthetic pathways.

It is structurally complex and has various forms depending on its building blocks (Nosanchuk et al., 2015; Choi et al., 2018). The mechanism of melanin synthesis varies depending on the radical formation; it can be synthesized through the random polymerization of a few building blocks, such as L-tyrosine metabolites of indole-5,6-quinone, 5,6-dihydorxyquinone carboxylic acid, 5,6-dihydroxyindole carboxylic acid (DHICA), dopamine, dopamine-o-quinone, homogentisate, cysteinylopa, and some phenolic precursors (Figure 1A) (Li et al., 2019; Seo and Choi, 2020b). Depending on the polymerization pathways, building blocks, and enzymes, melanin is classified into several groups, including eumelanin, pyomelanin, pheomelanin, neuromelanin, and allomelanin (Figure 1B) (Powell et al., 2004; Simon and Rozanowska, 2008).

The characteristic features of melanin vary depending on the class. The most common type of eumelanin consists of dihydroxyindole and DHICA, shows brown to black coloration, and can be produced by several microorganisms, including bacteria and fungi (Kuzumaki et al., 1993). Melanogenesis in the human skin, which is initiated by UV exposure, can lead to the formation of skin melanin and yellowish pheomelanin constitutes in the human skin (Wood and Schallreuter, 2006; Wolnicka-Glubisz et al., 2012; Pukalski et al., 2020). In addition, several catechol moieties have been reported to be involved in allomelanin production and can be found in plants and fruits (Varga et al., 2016; McCallum et al., 2021). Neuromelanin, which can be found in the brain, plays a critical role in treating neurodegenerative disorders (Usunoff et al., 2002; Double et al., 2008; Bellinger et al., 2011; Zucca et al., 2017). Similarly, it has been reported that melanin is involved in several physiological functions and as a result, serious genetic disorders are induced unless melanin is properly produced (Frenk and Lattion, 1982; Schmidt et al., 2015; Suo et al., 2020).

Melanin has been applied in a variety of biological, physiological, and physical materials, despite its complex random polymeric structures, which are responsible for its unique properties and functionality. Accordingly, great efforts have been made to screen melanin-producing strains for melanin production. For example, the isolation of melanin-producing fungal strains and the production of melanin on a large scale have attracted great attention. However, production titers and isolation methods vary depending on the host strain and melanin type. Although fungal strains are good hosts for melanin production, they require a long fermentation period to obtain the desired production titer (Nosanchuk et al., 2015; Cordero and Casadevall, 2017). In addition, the extraction and purification steps differ depending on the physical properties, such as solubility and the use of the isolated melanin (Pralea et al., 2019; Singh et al., 2021). Bioprocesses in melanin biorefinery include fermentation and extraction processes, which are widely used for biochemical production processes. Also, the treatment process of chemicals such as organic solvent in melanin extraction is included in the melanin biorefinery. Therefore, it is necessary to understand melanin production concerning the biorefinery process.

Several in-depth reviews on the chemical structure, engineering and production, and applications of melanin are available. For example, recent review articles by Pralea et al. and Singh et al. comprehensively reviewed the recent advances in melanin from biosynthesis to the application (Pralea et al., 2019; Park S. et al., 2020; Singh et al., 2021). In this review article, we summarize and highlight melanin bioproduction, including the current status of microbial production, extraction, and purification. In particular, we review the production of melanin from the space-time yield viewpoint, extraction from crude material, and pure melanin preparations. This review shares information on melanin biorefineries and supports the further development and potential applications of melanin.

## Bioconversion of Biomass Into Melanin

### Natural Melanin Sources and Alternatives

There is a variety of melanin sources; several common fruits and vegetables, such as apples, bananas, garlic, persimmons, and potatoes, can produce melanin (Lefevre and Perrett, 2015; Hagiwara et al., 2016; Qi et al., 2020). Melanin can also be obtained from plants, such as *Mucuna monosperma* (Wight) callus (Inamdar et al., 2014). Commercial melanin is prepared from sepia extract or by synthetic means (Prados-Rosales et al., 2015; Schroeder et al., 2015; Srisuk et al., 2016).

Nevertheless, these methods have the disadvantage of high production costs, low maneuverability, and environmental pollution risk. Therefore, the bioproduction of melanin by microorganisms such as fungi and bacteria as alternative melanin sources has attracted great attention. As they grow fast relatively and can be applied to the scale-up process for mass production. Besides, several attempts have been made to isolate melanin-producing strains from various environments to enhance melanin production through reactions and host cell engineering.

### Melanin Production by Fungal Strains

To date, several melanin-producing fungal strains have been reported (Rosas et al., 2000; Gomez et al., 2001; Nosanchuk et al., 2002; Morris-Jones et al., 2003; da Silva et al., 2006; Franzen et al., 2006; Nosanchuk et al., 2007; Walker et al., 2010; Nosanchuk et al., 2015). While fungal strains produce different types of melanin, the predominant melanin type is nitrogen-deficient allomelanin (Varga et al., 2016). The key enzymes responsible for melanin synthesis are tyrosinases, which are copper-dependent biocatalysts involved in ortho-specific hydroxylation and subsequent oxidation of monophenols like tyrosine (Kuzumaki et al., 1993). Laccase is another enzyme, which can catalyze the oxidation of a broad range of substrates like tyrosinase, including dihydroxyphenols and quinones (Nagai et al., 2003). Both enzymes are commonly abundant in plants and fungi rather than in bacteria. Therefore, fungal strains were potential candidate for melanin production. Moreover, the complex and dynamic membrane structure of fungi supplies a more suitable environment for melanin synthesis and deposition. For example, *Cryptococcus neoformans* melanin was reported to be located within the cell walls of branched polysaccharides and protein constructs (Nosanchuk and Casadevall, 2003). In addition, the presence of other cellular organizations, such as fungal vesicles, melanosomes, and anchoring structures, have been reported to assist in the efficient production and localization of fungal melanin (Nosanchuk and Casadevall, 2003; Nosanchuk et al., 2015; Camacho et al., 2019).

It should be noted, however, that the production of melanin-consuming fungus requires a relatively long incubation time due to the low cell growth rate of the fungus; for example, *Auricularia auricula* or *Gliocephalotrichum simplex* produced 2.97 g/L and 6.6 g/L of melanin in 8 and 6 days, respectively (Jalmi et al., 2012; Sun et al., 2016). Interestingly, Ribera et al. reported that 161 days of *Armillaria cepistipes* culture could produce 27.98 g/L of eumelanin, which was the highest as far as our understanding, in a 3% (w/v) tyrosine-supplemented medium (Ribera et al., 2019). However, it took a long time of 161 days to achieve this production titer.

It is possible to produce eumelanin from L-tyrosine and allomelanin via the polyketide pathway (Varga et al., 2016). However, limitations, such as low growth rate, sporulation, low extraction efficiency, and potential pathogenicity of fungal strains, need to be overcome to obtain desirable production titers. Recently, along with the development of genetic manipulation and sequencing technology, it has become possible to increase the productivity of various biochemicals with recombinant fungi through genetic engineering. In line with this, it could be possible to increase fungal melanin production through the expression of an external enzyme.

### Melanin Production by Bacterial Strains

Several microbial melanins have also been reported (Cubo et al., 1988; Jalmi et al., 2012; Ganesh Kumar et al., 2013; Surwase et al., 2013; Guo et al., 2014a; Madhusudhan et al., 2014; Tarangini and Mishra, 2014; Perez-Cuesta et al., 2020). Also, it was reported the production of melanin by using wild-type bacteria of *Klebsiella sp., Pseudomonas, Streptomyces, Bacillus, Amorphotheca,* and *Vibrio*, or by the expressing tyrosinase in *E. coli* as summarized in Table 1 (Tarangini and Mishra, 2014; Oh et al., 2020; Wang et al., 2020; Ahn et al., 2021)*.* Organisms with melanogenic capabilities have also been employed to develop production processes, which included production optimization of melanin by utilizing various carbon sources and culture variables; In particular, tyrosine, peptone, soy peptone, starch, and yeast extract were used as carbon sources or mixtures. This resulted in the biosynthesis of tyrosine-based eumelanin. The *Klebsiella sp*. GSK46 strain, which was isolated from crop field soil, was able to produce approximately 0.13 g/L of eumelanin when fed with 1 g/L of tyrosine (Sajjan et al., 2010).

**TABLE 1:** Production of microbial melanin in a biorefinery process.

No	Sources	Melanin type	Host strain	Genes expressed	Substrate (conc.)	Reaction condition	Reaction time	Production	Ref
1	Plant	—	*Mucuna monosperma (Wight) callus*	—	Tyrosine (1 g/L)	pH 5.5	48 h	0.887 g/L	Inamdar et al. (2014)
2	Fungus	—	*Auricularia auricula*	Wild type	Tyrosine (1.92 g/L), yeast extract (17.27 g/L), lactose (3.84 g/L)	pH 6, 28°C	8 days	2.97 g/L	Sun et al. (2016)
3	Fungus	Eumelanin	*Gliocephalotrichum simplex*	Wild type	Tyrosine (2.5% w/v), peptone (1% w/v)	28°C	6 days	6.6 g/L	Jalmi et al. (2012)
4	Fungus	Eumelanin	*Armillaria cepistipes*	Wild type	Tyrosine (3.0% w/v)	pH 6, 22°C	161 days	27.98 g/L	Ribera et al. (2019)
5	Bacterial	Eumelanin	*Klebsiella sp. GSK 46*	Wild type	Tyrosine (1 g/L)	pH 7.2, 37°C	3.5 days	0.13 g/L	Sajjan et al. (2010)
6	Bacterial	Eumelanin	*Pseudomonas stutzeri*	Wild type	Sea-water medium without tyrosine	pH 6.7, 37°C	10 h	6.7 g/L	Ganesh Kumar et al. (2013)
7	Bacterial	Eumelanin	*Streptomyces kathirae*	Wild type	Amylodextrine (3.3 g/L), yeast extract (5 g/L)	pH 6, 28°C	128 h	13.7 g/L	Guo et al. (2014a)
8	Bacterial	Eumelanin	*Bacillus safensis*	Wild type	Fruit waste extract	pH 6.84, 30.7°C	24 h	6.96 g/L	Tarangini and Mishra, (2014)
9	Bacterial	Eumelanin	*Streptomyces glaucescens NEAE-H*	Wild type	Protease-peptone (5 g/L)	30–37°C	6 days	3.16 g/L	El-Naggar and El-Ewasy, (2017)
10	Bacterial	Eumelanin	*Streptomyces sp. ZL-24*	Wild type	Soy peptone (20.31 g/L)	pH 7, 30°C	5 days	4.24 g/L (189.9 mg/L insoluble)	Wang et al. (2019)
11	Bacterial	Eumelanin	*Bacillus subtilis 4NP-BL*	Wild type	Starch (15 g/L)	pH 7.2, 28°C	7 days	1.5 g/L	Ghadge et al. (2020)
12	Bacterial	Eumelanin	*Escherichia coli*	*melC, cyp102G4*	Tyrosine, Indole	pH 7, 37°C	24 h	3.4 g/L	Park et al. (2020a)
13	Bacterial	Eumelanin	*Pseudomonas koreensis UIS 19*	Wild type	Molasses 5 Brix (5%), tyrosine (2.5 g/L)	pH 7.5, 30°C	24 h	5.5 g/L	Eskandari and Etemadifar, (2021)
14	Bacterial	Eumelanin	*Amorphotheca resinae*	Wild type	Peptone (10 g/L), yeast extract (5 g/L), glucose (20 g/L)	27°C	14 days	4.5 g/L (13.4 mg/L/h)	Oh et al. (2020)
15	Marine Bacterium	Eumelanin	*Vibrio natriegens*	*tyr1*	Tyrosine (0.4 g/L)	30°C	2 h	0.45 g/L (0.32 mg/mL/h)	Wang et al. (2020)
16	Bacterial	Pyomelanin	*Escherichia coli*	*4-hppd*	Tyrosine (1 mM)	pH 7, 37°C	6 days	0.213 g/L	Bolognese et al. (2019)
17	Bacterial	Pyomelanin	*Ralstonia picketti*	Wild type	Tyrosine (4 mM)	pH 7, 30°C	62 h	0.09 g/L	Seo and Choi, (2020a)
18	Bacterial	Pyomelanin	*Escherichia coli*	*4-hppd*	Tyrosine (4 mM)	pH 7, 30°C	24 h	0.315 g/L (13.1 mg/L/h)	Seo and Choi, (2020a)
19	Bacterial	Pyomelanin	*Yarrowia lypolytica* W29	4-HPPD	Tyrosine (1 g/L)	pH 7, 37°C	72 h	0.5 g/L	Ben Tahar et al. (2020)
20	Bacterial	Allomelanin	*Escherichia coli*	*fcs/ech*	Caffeic acid (5 mM)	pH 7, 37°C	12 h	0.2 g/L (40.9 mg/L/h)	Jang et al. (2018)
21	Bacterial	Allomelanin	*Escherichia coli*	*fcs/ech*	Caffeic acid (0.5 mM)	pH 7, 37°C	12 h	0.17 g/L (14.2 mg/L/h)	Ahn et al. (2019)

melC; tyrosinase from *Bacillus* megaterium, cyp102G4; cytochrome P450 monooxygenase from *Streptomyces* cattleya, 4-hppd; 4-hydroxyphenylpyruvate dioxygenase, tyr1; tyrosinase from *Bacillus* megaterium.

However, melanin could be produced even in the absence of tyrosine. For example, marine *Pseudomonas stutzeri,* isolated from seaweed, was found to produce significant amounts of melanin, which was 6.7 g/L within 10 h of incubation in sea water production medium without tyrosine supplementation (Ganesh Kumar et al., 2013). As fruit waste extract provides good nutrition for biochemical production, it has been utilized for melanin production. Tarangini and Mishra reported that *Bacillus safensis*, isolated from garden soil, could produce 6.96 g/L of melanin within 10 h of incubation (Tarangini and Mishra, 2014; Valdez-Calderón et al., 2020).

Amino acids also have been utilized for melanin production through whole cell biotransformation, in addition to the use of sugar-based fermentation including glucose, starch, and molasses (Ghadge et al., 2020; Oh et al., 2020; Eskandari and Etemadifar, 2021). For example, Eskandari and Etemadifar reported cost effective melanin production using *Pseudomonas koreensis* UIS19 in a molasses medium with tyrosine supplementation (Mustafa et al., 2020; Eskandari and Etemadifar, 2021). A total of 32 g/L of sugar was consumed to obtain 5.4 g/L of dry cell mass and 0.44 g dry melanin/g weight of yield could be achieved from supplemented tyrosine. In addition, several amino acid-based mediums, such as peptone and yeast extract, were utilized for melanin production using *Streptomcyes kathirae*, *Streptomyces glaucescens*, *Streptomyces* sp. ZL-24, and *Amorphoteca resinae*, which resulted in several g/L of melanin (Guo J. et al., 2014; El-Naggar and El-Ewasy, 2017; Wang et al., 2019; Eskandari and Etemadifar, 2021). In particular, melanin production by *S. kathirae* could reach up to 13.7 g/L, but 128 h of incubation was required for the highest titer (Guo J. et al., 2014).

It is worth noting that metal ions are critical for eumelanin production. For example, ferrous and nickel ion supplementation has been reported to drive melanin production by improving tyrosinase activity or by inducing the synthesis of tyrosinase (Wang et al., 2019). According to optimization results, 1.33 g/L FeSO_4_ and 3.05 mM NiCl_2_ could produce approximately 189.9 mg/L of insoluble melanin and 4.24 g/L of soluble pure melanin. The supplementation of metal ions seemed to have a positive effect on the activation of melanin production; however, the produced melanin was also reported to be able to chelate or absorb metal ions, such as Cu(II) and Zn(II), which would result in a metal-melanin complex and affect its characteristic features (Caldas et al., 2020; He et al., 2020).

### Pyomelanin and Allomelanin Production by Bacterial and Recombinant Strains

Another interesting type of melanin produced by bacteria is pyomelanin. Pyomelanin utilizes different synthetic pathways compared to bacterial eumelanin, even though they both are originated from L-tyrosine. The key enzyme in pyomelanin synthesis is the 4-hydroxyphenylpyruvate dioxygenase (4-HPPD) enzyme, which converts 4-hydroxyphenylpyruvate, a transaminated form of tyrosine, into homogentisate, a key intermediate in pyomelanin synthesis (Figure 1B). Recently, *Ralstonia picketti* was isolated and identified as capable of generating pyomelanin in the presence of tyrosine (Seo and Choi, 2020a). In the presence of 4 mM of tyrosine, *R. picketti* could produce about 0.09 g/L of pyomelanin within 62 h of incubation. To verify 4-HPPD-dependent pyomelanin synthesis, the encoding gene was isolated and cloned into *E. coli* BL21 (DE3). And the 4-HPPD overexpressing cells could produce 0.213 g/L of pyomelanin from 1 mM tyrosine within 24 h of incubation, suggesting that recombinant strain development could greatly enhance the production rate and titer (Seo and Choi, 2020a). Similarly, Bolognese et al. isolated the 4-HPPD enzyme and constructed a pyomelanin-producing recombinant *E. coli* strain that could produce 0.213 g/L of pyomelanin (Bolognese et al., 2019). In addition to bacterial strains, the yeast strain *Yarrowia lypolytica* W29 was isolated and verified to be capable of producing 0.5 g/L of pyomelanin by 1 g/L of tyrosine feeding (Ben Tahar et al., 2020). However, similar to fungal melanin production, a 72 h of incubation period was required to achieve the highest titer.

The development of a recombinant strain to produce allomelanin has also been extensively studied. For example, caffeic acid-based allomelanin production was investigated by our group. Jang et al. first reported the co-expression of feruloyl-CoA synthetase (FCS) and enoyl-CoA hydratase/aldolase (ECH) in an *E. coli* strain that drives allomelanin production in the presence of caffeic acids (Jang et al., 2018). These enzymes have been previously utilized in vanillin synthesis from ferulic acid (Gallage et al., 2014). As caffeic acid has a catechol moiety in its core structure, contrary to ferulic acid of which one hydroxyl group was blocked by the methoxyl group, the enzymatic modification of the other carboxylic moiety could readily lead to the formation of allomelanin. The FCS/ECH overexpressing recombinant strain could produce 0.2 g/L of allomelanin within a 12 h reaction (∼40.9 mg/L/h) (Jang et al., 2018). Ahn et al. also used the same strain to produce caffeic acid-based allomelanin and compared its chemical composition with that of other natural and synthetic melanin (Ahn et al., 2019). Interestingly, the caffeic acid-derived allomelanin showed substantial dyeing of the HEMA (hydroxyethyl methacrylate) polymer, which is generally used for soft contact lenses, suggesting the potential application of melanin as a UV-blocking contact lens.

Recombinant strains for melanin synthesis have several advantages, not only in terms of production rates and titers but also regarding extraction and purity. This was evident in studies conducted on the production of eumelanin and pyomelanin (Jang et al., 2018; Ahn et al., 2019; Bolognese et al., 2019; Park H. et al., 2020; Seo and Choi, 2020a). Another advantage is the possibility of an additional supply of melanin building blocks to control melanin chemical structure, this allows for the engineering of functionalities depending on the purpose of use. For example, we reported eumelanin engineering by co-expressing bacterial tyrosinase (MelC) with cytochrome P450 monooxygenase (CYP102G4), which is capable of catalyzing indole C2 hydroxylation (Park H. et al., 2020). The additionally supplied 2-hydroxyindole functioned as a new building block in melanin polymerization and could obtain different physical and electrical characteristics. However, several issues regarding the use of recombinant strains for melanin production should be addressed. For example, there is an issue regarding the safety of genetic engineering for use in cosmetics and pharmaceuticals. In addition, the dependency of the macroscopic structures and physical properties on the producing host should be considered.

### Understanding the Space-Time Yield of Melanin Bioproduction

**FIGURE 2:**
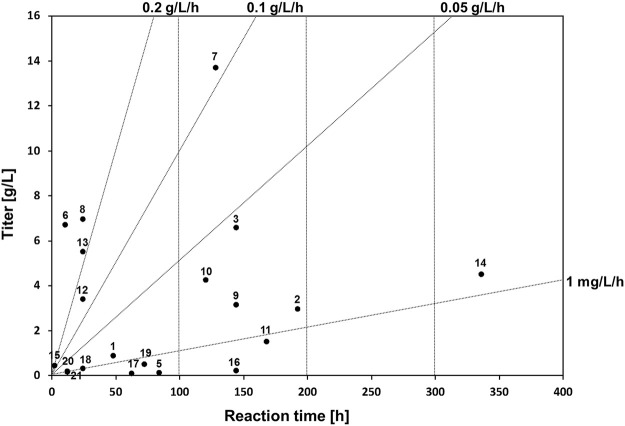
Space-time yield analysis of whole-cell biotransformation in melanin production and reported bioreactor scale by biotransformation yields. The numbers next to the circles indicate the corresponding reference numbers in Table 1.

To understand the reaction time and titer correlation in melanin production, a space-time yield analysis was conducted. Space-time yield analysis of the summarized microbial melanin production in g/L (closed circle, •) revealed that this biotransformation exhibited not distinct but observable two classes, namely those with a production rate range of less than 0.05 g/L/h and those with a range over 0.1 g/L/h (Figure 2). The first group includes most eumelanin- and pyomelanin-producing bacteria with less than 100 h of reaction time. The second group includes fungi and some *Streptomyces* species with more than 100 h of reaction time (Guo J. et al., 2014; Ribera et al., 2019). In particular, *A. auricula* showed the longest reaction time of 8 days with a moderate production titer (2.97 g/L) (Sun et al., 2016).

The second group had a relatively higher production rate. This group included several bacteria, such as *P. stutzeri*, *P. koreensis*, and *B. safensis,* which could produce more than 5 g/L of melanin within 24 h (Ganesh Kumar et al., 2013; Tarangini and Mishra, 2014; Eskandari and Etemadifar, 2021). Interestingly, melanin production by *S. kathirae,* with a 13.7 g/L titer within 128 h also belongs to this group, as it showed a production rate of more than 0.1 g/L/h (Guo J. et al., 2014). Compared to the fungal host system, the bacterial host system for whole-cell melanin production appears to be advantageous in terms of timescale, depending on the type of target melanin. However, several hurdles must be overcome to utilize bacterial hosts for industrial-scale production. One of the most limiting factors is the necessity of isolation and purification steps in circumstances where the synthesized melanin is not secreted. In addition, an adequate growth medium needs to be optimized to obtain a desirable cell mass.

In general, the pH for fungal melanin production was less than 6, whereas it was approximately neutral in bacterial cases. Although a pH of less than 7 was adopted for bacterial melanin production regarding *P. stutzeri*, *S. kathirae*, and *B. safensis*, which showed more than 5 g/L of production titer, the optimal pH for melanin production varied depending on the host cells (Ganesh Kumar et al., 2013; Guo J. et al., 2014; Eskandari and Etemadifar, 2021). The temperature for melanin synthesis is approximately 28°C for fungi and 30–37°C for bacterial systems. However, there seems to be no significant correlation between melanin production and temperature; rather, it seems more important to secure the maximal cell mass for melanin production under optimized conditions. Therefore, melanin production should be focused on the optimization of production parameters, such as growth medium composition, pH, temperature, extraction parameters, in addition to the design of response surface methodology in order to obtain a higher production titer and rate.

## Bioprocess for Melanin Production; Fermentation, Extraction, and Purification

### Extraction of Melanin From Melanin Production Culture

**FIGURE 3:**
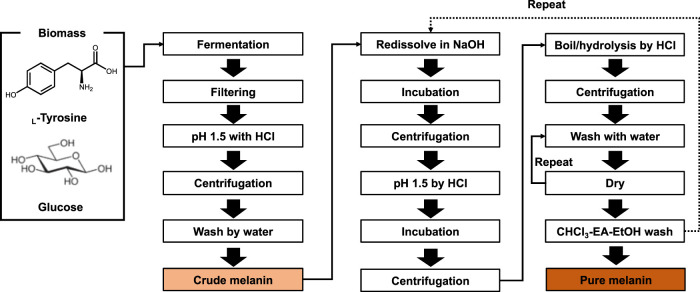
Bioprocess of melanin production and isolation. By several steps of purification, crude and pure melanin can be obtained.

The basic melanin production process includes host selection, fermentation or biotransformation, followed by securing crude melanin through extraction and purification processes to obtain pure melanin (Figure 3). The method of extracting melanin differs depending on the host cell that is producing melanin, the intracellular localization of melanin, the structural properties of melanin, and the melanin crystal structure. As melanin pigment can easily be found in nature, research on extracting melanin was conducted early on (Aneesh et al., 2020). In particular, methods for extracting melanin pigment from melanocytes and melanin organs, which are generally extracted by dissolving in an alkali or strong acid solution and heating (Young, 1921; Voss, 1954). For example, crude melanin was obtained by simple alkali extraction; however, the yield was as low as 2.59% (Ma et al., 2018). The extraction and purification process of melanin affects the purity of melanin, depending on the extraction method, the number of repeat cycles, and the form of melanin, namely crude or pure melanin.

### Preparation of Crude Melanin From Melanin Extract

The detailed extraction process for microbial melanin production is presented in Table 2. Depending on the melanin source, it is divided into extracellular and intracellular melanin. Extracellular melanin extraction methods employ acid precipitation, whereas additional alkali extraction is necessary for intracellular melanin production. To assist alkali extraction, ultrasonic- or microwave-assisted methods were used (450 W for 50 min, or 70 W for 3 min periods with 30 cycles) (Sajjan et al., 2010; Jalmi et al., 2012; Lu et al., 2014; Hou et al., 2019; Liu et al., 2019). According to Hou et al., the ultrasonic-assisted extraction method yielded 37.33% pure melanin, whereas 24.24% was obtained without this step (Hou et al., 2019). Similarly, Lu et al. reported that a purification yield of 11.08%% could be achieved through a microwave-assisted extraction method, which was 40.43% higher than that obtained by alkali extraction and acid precipitation. In addition, an additional step of boiling at 80°C for 2 h was employed to increase the extraction yields (Oh et al., 2020).

**TABLE 2:** Extraction and purification process details in melanin biorefinery.

Sources	Sources	Melanin type	Extraction methods	Performance	Ref
Mushroom	*Auricularia auricula-judae*	—	69.11°C, 58.66 min, pH 12.81	2.59% yield	Ma et al. (2018)
Mushroom	*Inonotus hispidus*	—	Sample to liquid (1:50) in NaOH (0.56 mol/L) with ultrasonic waves-assisted extraction (300 W, 70°C, 70 min), collect by centrifugation (10,000 rpm, 10 min)	37.33% yield (24.24% yield w/o ultrasonic)	Hou et al. (2019)
Mushroom	*Auricularia auricula*	—	Crude melanin: sample to liquid (1:44) in NaOH (0.58 mol/L) with ultrasonic waves-assisted extraction (450 W, 70°C, 50 min), collection by centrifugation, adjust pH to 1.5 with HCl (6 mol/L), incubate at 80°C for 10 h, collect by centrifugation, and wash with deionized water Pure melanin: re-dissolve in NaOH (1.5 mol/L), collect by centrifugation (10,000 rpm, 5 min), adjust pH to 1.5 with HCl (6 mol/L), incubate at 4°C for 5 h, collect by centrifugation (10,000 rpm, 5 min), wash with deionized water to pH 7, wash with CHCl_3_, DCM, EA, and EtOH, followed by freeze-drying	11.99% yield	Liu et al. (2019)
Mushroom	*Lachnum singerianum* YM296	—	NaOH concentration, 1.05 mol/L; ratio of raw material to liquid ratio, 1:14.72 (g/ml); microwave time, 118.70 s; and microwave power, 320 W	11.08% yield	Lu et al. (2014)
Fungus	*Gliocephalotrichum simplex*	Eumelanin	Collect by centrifugation (12,000 g, 15 min), filter using 0.22 μm membrane filters, precipitate using acetic acid (1 M), collect by centrifugation and wash, dry and resolubilize in NaOH (0.1 M, pH 12), adjust pH by HCl (0.1 M)	6.6 g/L	Jalmi et al. (2012)
Fungi	*Amorphotheca resinae* KUC3009	Eumelanin-like	Crude melanin: filter through 0.45-μm glass fiber, mix with NH_3_·H_2_O (1 M, 1:1 v/v), boil at 80°C for 2 h, adjust pH to 2 with HCl (6 M), incubate at 21°C for 24 h, and collect by centrifugation Pure melanin: resuspend in HCl (6 M), boil at 100°C for 4 h, rinse repeatedly with deionized water, re-dispersed in deionized water, extract with CHCl_3_, EA, and EtOH, and lyophilize	4.5 g/L (13.4 mg/L/h)	Oh et al. (2020)
Fungi	*Auricularia auricula*	Eumelanin	Crude melanin: squeeze through a nylon mesh, adjust pH 1.5 with HCl (6 mol/L), store overnight at 4°C, collect by centrifugation (10,000 rpm, 15 min, at 4 °C), wash with deionized water, and dry Pure melanin: redissolve in NaOH (2 mol/L), stir overnight, collect by centrifugation (10,000 rpm, 15 min, at 4°C), adjust pH to 1.5 by HCl (7 mol/L), incubate at room temperature for 2 h, collect by centrifugation (10,000 rpm, 15 min, at 4°C), hydrolyze with HCl (7 mol/L) at 100°C for 2 h, collect by centrifugation (10,000 rpm, 15 min, at 4°C), wash three times with distilled water, dry and wash with CHCl_3_, EA, EtOH, dry at room temperature, redissolve in NaOH (2 mol/L), collect by centrifugation (10,000 rpm, 15 min, at 4°C), adjust pH to 1.5, collect by centrifugation (10,000 rpm, 15 min, at 4°C), repeat and dry at 60°C	2.97 g/L	Sun et al. (2016)
Fungi	*Armillaria cepistipes*	Eumelanin	Raw melanin: filter through a 0.45 μm nitrocellulose membrane, sterilize by autoclaving (20 min, 121°C, 1 bar), and lyophilize (28.0 g/L melanin powder) Pure melanin: adjust pH to 2 by HCl (5 M), collect by centrifugation (8,000 rpm, 15 min), wash with water using four cycles of centrifugation−redispersion (until pH ∼6 is reached, vortex, and sonicate), wash with EtOH three times, ethanolic suspension, and lyophilize	17.0 g/L pure melanin from 28.0 g/L raw melanin	Ribera et al. (2019)
Bacterial	*Pseudomonas koreensis UIS 19*	Eumelanin	Crude melanin: collect by centrifugation (2,600 g, 15 min), wash by EtOH-acetone (1:1) solution, collect by centrifugation (2,600 g, 15 min), boil for 15 min, and dry	0.44 g dry wt/g L-tyrosine	Eskandari and Etemadifar, (2021)
Bacterial	*Streptomyces kathirae*	Eumelanin	Collect by centrifugation (5,000 g, 15 min), adjust pH to 3, resuspend in HCl (6 M) for 4 h, collect by centrifugation (5,000 g at room temperature), adjust pH to 9 and 3 twice for precipitation, wash with distilled water, collect by centrifugation, and dry at 70°C	13.7 g/L	Guo et al. (2014a)
Bacterial	*Streptomyces glaucescens NEAE-H*	Eumelanin	Collect by centrifugation (5,000 g, 15 min), adjust pH to 2 by HCl (6 M) for 4 h, collect by centrifugation (9,000 g, 15 min), wash with distilled water for four times, collect by centrifugation (9,000 g, 15 min), and lyophilize	0.35 dry wt/L	El-Naggar and El-Ewasy, (2017)
Bacterial	*Vibrio natriegens*	Eumelanin	Collect by centrifugation, filter through a Millipore 0.2-μm polyether sulfone membrane, precipitate by HCl (6 N, 10% v/v), wash with deionizing water until neutral pH, and lyophilize	66 mg mel/g CDW/h	Wang et al. (2020)
Bacterial	*Bacillus subtilis 4NP-BL*	Eumelanin	Collect by centrifugation (6,720 g, 15 min), adjust pH 2 by HCl (6 M), precipitate for 4 h, collect by centrifugation (10,510 g, 15 min), wash with distilled water for four times, collect by centrifugation (10,510 g, 15 min), and vacuum-dry	1.5 g/L	Ghadge et al. (2020)
Bacterial	*Bacillus safensis*	Eumelanin	Collect by centrifugation (9,200 g, 15 min), suspended in distilled water, collect by centrifugation, adjust pH to 2 by HCl (3 N), incubate for 48 h at RT, repeat, boil for 5 min, and collect by centrifugation (4,600 g, 15 min)	6.96 g/L	Tarangini and Mishra, (2014)
Bacterial	*Amorphotheca resinae*	Eumelanin	Collect by centrifugation, filter using 0.45-μm glass fiber, mix with NH_3_·H_2_O (1 M, 1:1 v/v), boil at 80°C for 2 h, adjust pH to 2 by HCl (6 M), incubate for 24 h at 21°C, collect by centrifugation, resuspend in HCl (6 M), boil at 100°C for 4 h, rinse repeatedly with deionized water, re-disperse in deionized water, extract with CHCl_3_, EA, and EtOH, and lyophilize	4.5 g/L	Oh et al. (2020)
Bacterial	*Klebsiella sp. GSK 46*	Eumelanin	Disrupt by Vibracell ultrasonicator in an ice bath (70 W, 30 cycles of 3 min, 1 min off per cycle), adjust pH to 2 by HCl (1 N), boil for 1 h, collect by centrifugation (8,000 g, 10 min), wash three times with HCl (0.1 N, 15 ml) and with water, mix with EtOH (10 ml), boil for 10 min, incubate at room temperature for 24 h, wash with EtOH two times, and dry in air	0.13 g/L	Sajjan et al. (2010)

Before acid precipitation, filtering through various materials, such as 0.45 μm glass fiber, 0.45 μm nitrocellulose membrane, 0.22 μm membrane filter, and Millipore 0.2 μm polyether sulfone membrane, were employed to remove cell debris and byproducts (Jalmi et al., 2012; Ribera et al., 2019; Oh et al., 2020; Wang et al., 2020). To assist precipitation, a boiling or incubation step for several hours may be added (Sajjan et al., 2010; Sun et al., 2016; Liu et al., 2019). After precipitation, a washing step with deionized water was conducted. Crude melanin could be prepared using these filtration-precipitation-centrifugation-washing procedures (Figure 3).

### Preparation of Pure Melanin Powder From Crude Melanin

To increase the purity of the isolated crude melanin, several redissolution, precipitation, boiling, and washing steps were employed. In brief, crude melanin was dissolved in NaOH and collected by centrifugation. Thereafter, the pH of the collected sample was adjusted to approximately pH 2 with HCl, followed by incubation. The resuspended melanin was collected by centrifugation and washed several times with deionized water. Finally, the collected melanin was washed with CHCl_3_, DCM, EA, and pure EtOH, followed by lyophilization. Depending on the melanin type and condition, extra boiling, acid-hydrolysis, and repetitive washing steps can be added to pure melanin (Sun et al., 2016; Liu et al., 2019; Ribera et al., 2019; Oh et al., 2020).

Several simplified extraction methods have been proposed, however, acid precipitation-pH adjustment-washing-resuspension steps are commonly used (Sajjan et al., 2010; Jalmi et al., 2012; Guo J. et al., 2014; Tarangini and Mishra, 2014; Ghadge et al., 2020; Wang et al., 2020). In addition, other useful technologies have been applied to melanin extraction. For example, enzymatic disruption of the cell membrane using protease or hydrolase enzymes, instead of alkali extraction, has been utilized. Additionally, a variety of organic solvents has been utilized for melanin extraction with excellent yields. With respect to bioprocess, an issue regarding environmental concerns should be considered as such use of organic solvent and effluent disposal for melanin isolation process. As described above, several extraction methods could be applied to different types of melanin and different sources, suggesting that no optimal method can be applied consistently. Depending on the chemical structure, type, solubility, and purpose of use, it seems appropriate to use optimized methods specific to each process.

## Limitations of Microbial Melanin in Commercialization and Industrial Uses

Despite its high potential as functional biomaterials, its commercialized use has been limited. One possible explanation for that is due to its complexity and diversity in commercialization. As the synthetic route includes radical-based random organization, keeping and controlling of physical properties and biological functionalities consistently is difficult. And this also relates to the difficulties in standardization of melanin quality and performance. Also, as it is produced by microorganisms, whether genetically modified or not, it is limited due to various regulations regarding human toxicity to be applied to physiologically active materials targeting the human body. Along with the harmful of toxic microorganisms, the use of strong acid/base and organic solvent of DCM, EA, and chloroform is surely a burden in effluent disposal and one of the limitations in the commercialization of microbial melanin, and this should be overcome by the engineering of isolation process with eco-friendly manner.

Besides, economics for the melanin production process should be considered. In industrial applications, materials that can produce several g/L of end products with wild-type strains are unusual even if no special genetic engineering is applied. In the biotransformation process, it is critical for the tyrosine conversion reaction to secure an adequate cell mass and increase the activity of conversion enzymes, such as tyrosinase, laccase, and 4-HPPD. In addition, it is important to increase the production yield of melanin to ensure a competitive market price of melanin using tyrosine as a substrate, as tyrosine has a higher market price than sugar-based biomass. Alternatively, another solution could be the intracellular supply of tyrosine through metabolic engineering from cost-effective carbon sources. The application of melanin to high-value-added fields should also be considered.

## Application of Melanin and Future Perspectives

Due to its black pigmentation in the skin, inhibition of melanin formation by tyrosinase inhibitors has been focused on for a long time. Such inhibitors are often utilized as ingredients in skin-whitening cosmetics (Cabanes et al., 1994; Netcharoensirisuk et al., 2021). However, interesting features of melanin, including its ability as a UV protector, a radical scavenger, and a chelator against metal ions, have driven melanin production as a functional material with promising cosmetic, pharmaceutical, and environmental applications. In addition, the electron-storing capacity of melanin has enabled its application as an electrode and supercapacitor (Park H. et al., 2020; Hernández-Velasco et al., 2020).

As such, a pigment made by microorganisms is receiving substantial focus. In particular, the use of bio-pigments with biocompatibility can be used in various fields, such as cosmetics, medicine, pharmaceuticals, and the environment. Highlighting the industrial application of melanin, recently various products have been released in the beauty field of hair care, such as dyeing and shampooing, which utilize water-soluble squid melanin (Aghajanyan et al., 2005; Guo X. et al., 2014; Longo et al., 2017).

As a strategy for securing both the productivity and the high value-added application of melanin in biological processes, the simultaneous production of melanin and biochemicals in a single cell could also be used. For example, Ahn et al. recently reported the co-production of melanin with valuable biochemical, such as cadaverine, which is a diamino pentane obtained from the decarboxylation of lysine (Ahn et al., 2021). According to the study, the produced cadaverine was directly incorporated in melanin polymerization. This co-production process would be a solution to ensure a competitive market price. From a bioprocess point of view, it is appealing to produce biochemicals with such functionality only by single enzyme expression. Research on obtaining excellent functionality through additional building block-based structural modifications in recombinant melanin-producing strains should also be in the spotlight in the future.

## Conclusion

Melanin is the pigment that is most frequently encountered and is one of the constituents of human skin tissue. Melanogenesis is possible in various organisms, and its mass production has become possible through the discovery of melanin-producing microorganisms and bioconversion processes. As a result of the study on the complex melanin chemical structure and physicochemical properties, melanin extraction, separation, and purification optimization studies have been conducted. Through these studies, crude melanin and pure melanin can be produced at a level of several g/L. In the biochemical field, there is still a need for research on increased productivity at the level of space-time yields that can be matched in the fine chemical and pharmaceutical industries. Above all, two of the key limitations that need to be overcome for the industrial application of melanin are securing the substrate and securing price competitiveness in the bioconversion process of the tyrosine substrate. In addition, the link between biological function and structural complexity of melanin needs to be better understood to fully reproduce the functional properties of melanin, allowing for its development as an actual biochemical product.
